# Changes in Patient-Reported Outcome Measures With a Technology-Supported Behavioral Lifestyle Intervention Among Patients With Type 2 Diabetes: Pilot Randomized Controlled Clinical Trial

**DOI:** 10.2196/19268

**Published:** 2020-07-24

**Authors:** Rozmin Jiwani, Jing Wang, Andrea Berndt, Padmavathy Ramaswamy, Nitha Mathew Joseph, Yan Du, Jisook Ko, Sara Espinoza

**Affiliations:** 1 University of Texas Health Science Center at San Antonio San Antonio, TX United States; 2 Cizik School of Nursing The University of Texas Health Science Center at Houston Houston, TX United States

**Keywords:** Patient-Reported Outcome Measurement Information System, patient-reported outcomes, patient-reported outcome measures, type 2 diabetes, self-management, self-monitoring, behavioral lifestyle interventions

## Abstract

**Background:**

In the United States, more than one-third of the adult population is obese, and approximately 25.2% of those aged ≥65 years have type 2 diabetes (T2D), which is the seventh leading cause of death. It is important to measure patient-reported outcomes and monitor progress or challenges over time when managing T2D to understand patients’ perception of health and quantify the impact of disease processes or intervention effects. The evaluation of patient-reported outcome measures (PROMs) is especially important among patients with multiple chronic conditions in which clinical measures do not provide a complete picture of health.

**Objective:**

This study examined the feasibility of collecting Patient-Reported Outcome Measurement Information System (PROMIS) measures, and preliminarily evaluated changes in PROMIS scores and compared the scores with standard scores of the general US population. The parent study is a pilot randomized controlled clinical trial testing three different modes (mobile health [mHealth], paper diary, and control) of self-monitoring in a behavioral lifestyle intervention among overweight or obese patients with T2D.

**Methods:**

Patients with comorbid overweight or obesity and a diagnosis of T2D for at least 6 months were recruited from a diabetes education program. Participants were randomized to the following three groups: mHealth, paper diary, and control (standard of care) groups. Paper diary and mHealth experimental groups received additional behavioral lifestyle intervention education sessions, as well as tools to self-monitor weight, physical activity, diet, and blood glucose. All participants completed PROMIS-57 and PROMIS-Global Health (GH) version 1.0 questionnaires during visits at baseline, 3 months, and 6 months. The PROMIS-57 includes the following seven domains: anxiety, depression, fatigue, pain interference, physical function, satisfaction with participation in social roles, and sleep disturbance. The PROMIS-GH is composed of the following two domains: global mental health and global physical health.

**Results:**

A total of 26 patients (mHealth, 11; paper diary, 9; control, 6) were included in our analysis. The study sample was predominantly African American (68%) and female (57%), with a mean age of 54.7 years and a mean BMI of 37.5 kg/m^2^. All patients completed the PROMIS-57 and PROMIS-GH questionnaires, and we compared the mean scores of the three groups to investigate potential differences. No relevant differences were noted across the groups. However, positive trends were noted in both intervention (mHealth and paper diary) groups in the middle (month 3) and end (month 6) of the study.

**Conclusions:**

Our pilot study provides evidence for the feasibility of using PROMIS questionnaires to record important components of T2D-related symptoms among overweight or obese individuals. The results from our study support the use of PROMIS questionnaires to provide clinicians and researchers with a benchmark for assessing the overall need for symptom management and determining the success or challenges of an intervention.

**Trial Registration:**

ClinicalTrials.gov NCT02858648; https://clinicaltrials.gov/ct2/show/NCT02858648

## Introduction

In the United States, more than one-third of the adult population is obese [1], and the prevalence of obesity among adults in Texas is currently 33.0% (up from 21.7% in 2000). Texas is ranked 14th for obesity in the United States [2]. Obesity-related conditions, such as type 2 diabetes (T2D), heart disease, stroke, and certain types of cancer, are some of the leading causes of preventable premature death [3]. Obesity affects some groups more than others, and Hispanic people and non-Hispanic black people were reported to have the highest age-adjusted prevalence of obesity at 47.0% and 46.8%, respectively [3]. T2D is the seventh leading cause of death in the United States, and the percentage of adults with diabetes shows an increase with age, reaching a high of 25.2% among those aged ≥65 years [4]. Compared with non-Hispanic white people, the age-adjusted prevalence of diagnosed and undiagnosed diabetes was reported to be higher among Hispanic and non-Hispanic black people [4]. The management of diabetes is complex, and people living with diabetes need to make many choices related to their treatment and management, including self-monitoring their diet and activity.

Mobile health (mHealth), defined as the delivery of health care services and information using mobile technologies, is being increasingly utilized in diabetes management [5,6]. The technologies include smartphones, wearable devices, smart and connected devices, and apps. Several studies and systematic reviews have strongly supported the effectiveness of mobile apps and smart devices for diabetes management in recent years [5-8]. The authors have highlighted the positive clinical outcomes of these interventions, including reduction of hemoglobin A_1c_, when compared with standard care, and other diabetes and cardiometabolic variables such as blood glucose levels, blood pressure, serum lipid levels, and body weight.

Although clinical outcomes are important in the management of T2D, it is also important to integrate patient-reported outcome measures (PROMs) to improve overall care among people with T2D. The Patient-Reported Outcome Measurement Information System (PROMIS) initiative from the National Institutes of Health was developed to improve and standardize measurements of patient-reported outcomes [9]. PROMIS is a set of person-centered measure scores that screen; evaluate interventions; and monitor physical, mental, and social health and well-being in general populations and among individuals with chronic conditions to understand their perceptions of health and ultimately what information is most meaningful to them [9]. This is especially important among patients with chronic conditions in which clinical performance measures do not provide a complete picture of health [10]. It can also provide clinicians and researchers with a benchmark for assessing the overall need for treatment and management, and determining the success or challenges of an intervention or treatment [9]. There is growing evidence of the validity of PROMIS tools, and they are in widespread use but have undergone limited validation in vulnerable populations with multiple comorbid conditions, including overweight or obese adults with T2D.

This study is a secondary analysis examining the feasibility of collecting PROMIS data and a preliminary evaluation of changes in PROMIS questionnaires, as part of a pilot randomized controlled clinical trial testing three different modes (mHealth, paper diary, and control) of self-monitoring of behavioral lifestyle interventions among overweight or obese patients with T2D [11]. We compared the mean scores and SDs for the three study groups to the data of a US reference population. The presence of a common set of outcome metrics would greatly improve the ability to compare outcomes across institutions and populations and inform the provision of effective care [9].

This study aimed to examine the feasibility of collecting PROMIS measures and to preliminarily evaluate the changes in PROMIS-57 and PROMIS Global Health (GH) questionnaires in a pilot randomized controlled clinical trial testing three different modes (mHealth, paper diary, and control) of self-monitoring of behavioral lifestyle interventions among overweight or obese adults with T2D from a parent study [11].

## Methods

### Recruitment

Participants were recruited from a certified American Diabetes Association diabetes education program in Harris County, Texas. The primary clientele of the selected location is uninsured or underinsured individuals from the surrounding area. Flyers were distributed by diabetes educators to patients attending diabetes education classes, and interested participants could contact the study team for additional details and enrollment.

Participants were screened for eligibility based on the following criteria: (1) diagnosis of T2D for at least 6 months (confirmed in the electronic health records); (2) overweight or obesity (BMI ≥25 kg/m^2^); (3) age 21 to 75 years; (4) ability to read and write in English; and (5) completion or near completion of the basic diabetes self-management education offered at the recruitment site. The exclusion criteria were as follows: (1) history of severe psychiatric disorders (eg, bipolar disorder and schizophrenia); (2) inability to perform regular activity; (3) current pregnancy or planning for pregnancy or nursing in the next 6 months; (4) planning for a vacation in the next 6 months; (5) previous participation in an intensive behavioral lifestyle intervention; and (6) substance abuse in the past year.

This paper describes the secondary data analysis of a three-group pilot randomized controlled clinical trial, which compared the efficacy of behavioral lifestyle interventions using either mHealth or paper diary self-monitoring tools among underserved populations with comorbid T2D and overweight or obesity [11]. The pilot study’s primary and secondary outcome measures were glycemic control and weight, respectively. All three groups completed usual diabetes care and education; the mHealth and paper diary experimental groups additionally completed 11 group sessions as part of the behavioral lifestyle intervention over 6 months and self-monitored their diet, physical activity, weight, and blood glucose levels [11].

In addition to other measures, participants in all three groups completed PROMIS-57 and PROMIS-GH version 1.0 questionnaires at baseline, 3 months, and 6 months to evaluate the impact of behavioral lifestyle interventions on PROMs in this study.

There is substantial clinically valid evidence that PROMIS was successful in developing measures that are effective across a range of chronic conditions (chronic heart failure, chronic obstructive pulmonary disease, rheumatoid arthritis, cancer, back pain, and major depression) and predominately in white non-Hispanic people [12]. The PROMIS-57 is intended for use across a variety of conditions and assesses the following seven domains: physical function, anxiety, depression, fatigue, sleep disturbance, pain interference, and satisfaction with participation in social roles and activities [13]. There is also a single item in all PROMIS questionnaires measuring pain intensity, which has 11 response options ranging in value from 0 to 10. For the pain intensity domain, higher values reflect greater pain [13]. The seven domains are composed of eight items, with response options ranging on a 5-point Likert scale (always, often, sometimes, rarely, and never). For the physical function and satisfaction with participation in social roles domains, higher scores reflect better functioning. In contrast, for the anxiety, depression, fatigue, pain interference, and sleep disturbance domains, higher scores reflect poorer functioning. PROMIS-GH refers to a person’s general evaluation of health and produces the following two scores: global physical health (GPH) and global mental health (GMH). The GPH assesses physical health (ie, physical functioning, pain intensity, and fatigue), whereas the GMH assesses overall quality of life, mental health, satisfaction with social activities, relationships, and emotional problems. Higher scores for GMH and GPH reflect better functioning [13].

### Statistical Analysis

Statistical Package for the Social Sciences (SPSS) version 25 (IBM Corp) was used for all statistical analyses. Significance was set at α=.05 [14]. For all variables, frequency distributions were generated. For nominal and ordinal variables, percentages and modes were evaluated. For interval and ratio variables, means and standard deviations were calculated if the variables were normally distributed. When response sets for participants were incomplete, missing responses were imputed using regression substitution if 80% or more of the responses were present. After imputations, raw score totals and T-scores were generated for complete response sets [13]. The Mann-Whitney *U* test was used when comparing two groups for an ordinal dependent variable (mHealth group and paper diary group). The Kruskal-Wallis test was used when comparing three groups (mHealth, paper diary, and control) for an ordinal dependent variable. To compare the T-scores of the mHealth group to those of the paper diary group for the seven domains from the PROMIS-57 and the two domains from the PROMIS-GH, Mann-Whitney *U* analyses were performed. To examine if the T-scores for the seven domains from the PROMIS-57 and the two domains from the PROMIS-GH varied as a function of the group at each time point, Kruskal-Wallis tests were performed.

## Results

### Sample Description

A total of 26 patients (11 in the mHealth group, 9 in the paper diary group, and 6 in the control group) were included in our analysis. The study sample consisted of predominantly African American (68%) and female (57%) participants, with a mean age of 54.7 years and a mean BMI of 37.5 kg/m^2^. A detailed description of the sample can be found in the parent study [11].

Table 1 presents the descriptive statistics for PROMIS-57 and PROMIS-GH. The feasibility of collecting PROMIS-57 and PROMIS-GH data in a pilot randomized controlled clinical trial is high, and all of the 26 patients completed the baseline and 3- and 6-month assessments on PROMIS tools.

The analysis compared group scores at baseline, 3 months, and 6 months. Evaluation of the results from the Mann-Whitney *U* test and Kruskal-Wallis test indicated that there were no relevant differences in PROMIS scores among the three groups at any time point. However, interpretation of the results from the Mann-Whitney *U* test indicated a trend for the PROMIS-GH domain GMH at 3 months (*U*=−1.8, *P*=.06), that is, GMH showed a trend of lower scores in the mHealth group (mean 42.3, SD 4.5) than in the paper diary group (mean 45.9, SD 5.2) at 3 months; however, this was not significant (*P*=.06).

**Table 1:** Descriptive statistics on PROMIS-57 and PROMIS Global Health.

Questionnaire domain and time point^a^				mHealth^b^ (n=11), mean (SD) score	Paper diary (n=9), mean (SD) score	Control (n=6), mean (SD) score
**PROMIS^c^-57**						
	**Physical Function^d^**					
			Baseline	40.6 (7.5)	39.1 (6.3)	41.5 (5.2)
		Three months		41.4 (8.4)	44.1 (7.9)	42.5 (8.9)
		Six months		40.2 (8.2)	47.1 (10.1)	45.1 (11.9)
	**Satisfaction with social roles^d^**					
			Baseline	46.6 (9.2)	42.4 (10.5)	46.5 (4.3)
		Three months		49.0 (10.3)	53.4 (11.9)	42.5 (7.5)
		Six months		45.6 (11.4)	46.6 (14.8)	52.0 (13.1)
	**Anxiety^e^**					
			Baseline	53.4 (10.2)	56.1 (11.6)	52.0 (10.5)
		Three months		54.8 (8.1)	55.0 (9.9)	51.6 (6.7)
		Six months		51.7 (11.7)	50.4 (14.2)	48.1 (8.5)
	**Depression^e^**					
			Baseline	48.9 (9.9)	53.2 (10.5)	45.0 (10.2)
		Three months		49.0 (9.8)	49.8 (11.6)	50.4 (3.0)
		Six months		46.6 (12.9)	49.9 (12.7)	44.0 (7.4)
	**Fatigue^e^**					
			Baseline	53.5 (10.1)	55.4 (12.6)	52.9 (5.5)
		Three months		54.0 (7.7)	53.1 (11.1)	53.5 (7.8)
		Six months		53.9 (7.7)	47.8 (13.1)	52.8 (2.9)
	**Sleep disturbance^e^**					
			Baseline	56.4 (5.4)	53.0 (10.0)	59.4 (3.1)
		Three months		56.6 (3.2)	56.1 (5.7)	59.3 (1.7)
		Six months		56.6 (5.6)	59.5 (4.1)	56.8 (4.4)
	**Pain interference^e^**					
			Baseline	61.0 (9.1)	57.0 (10.3)	59.5 (5.5)
		Three months		61.7 (5.2)	56.3 (11.8)	57.6 (11.5)
		Six months		58.2 (9.8)	55.1 (12.5)	57.2 (3.5)
	**Pain intensity^e^**					
			Baseline	5.7 (2.4)	4.7 (2.4)	4.5 (4.2)
		Three months		5.5 (2.8)	4.2 (3.1)	5.3 (4.4)
		Six months		4.8 (2.5)	3.6 (3.5)	5.6 (2.3)
**PROMIS-GH^f^**						
	**Global Physical Health^d^**					
			Baseline	37.8 (4.2)	39.3 (5.1)	39.4 (4.2)
		Three months		38.7 (4.0)	39.8 (5.0)	41.4 (7.0)
		Six months		38.2 (4.8)	41.1 (5.4)	42.5 (5.9)
	**Global Mental Health^d^**					
			Baseline	40.8 (4.4)	42.3 (4.5)	45.8 (2.6)
		Three months		42.3 (4.5)	45.9 (5.2)	45.5 (6.2)
		Six months		42.3 (5.1)	42.8 (6.0)	44.4 (4.0)

^a^For all domains listed, the mean (SD) score for the US reference population is 50 (10).

^b^mHealth: mobile health.

^c^PROMIS: Patient Reported Outcome Measurements Information System.

^d^Higher scores indicate better functioning.

^e^Higher scores indicate worse functioning.

^f^PROMIS-GH: Patient Reported Outcome Measurements Information System Global Health.

Thereafter, we compared the mean scores and SDs for the three groups (ie, mHealth, paper diary, and control) to the data of the US reference population. The mean score and SD for the US reference population for each of the seven domains of the PROMIS-57 and two domains of the PROMIS-GH were 50 and 10, respectively. For each domain, a higher score indicates that more of the concept has been measured. For the three groups in our study, the mean scores for physical function (PROMIS-57) and for PROMIS-GH were lower than the scores in the US reference population (mean score of 50), indicating poorer functioning (Table 1). Specifically, for physical function, the mean scores ranged from 39.1 to 47.1; for GMH, the mean scores ranged from 37.8 to 42.5; and for GPH, the mean scores ranged from 40.8 to 45.9. In addition, for the three groups in our study, the mean scores for anxiety, pain interference, and sleep disturbance were higher than the scores in the US reference population (mean score of 50), indicating poorer functioning. Specifically, for anxiety, the mean scores ranged from 48.1 to 56.1; for pain interference, the mean scores ranged from 55.1 to 61.7; and for sleep disturbance, the mean scores ranged from 53.0 to 59.5. Furthermore, the baseline scores of anxiety (mean 53.4), depression (mean 48.9), pain interference (mean 61.0), and pain intensity (mean 5.7) improved at the end of the study in the mHealth intervention group.

## Discussion

### Principal Findings

Our pilot study provides evidence for the feasibility of using the PROMIS questionnaires to measure patient-reported outcomes among overweight or obese individuals diagnosed with T2D. The mean T-scores across time for each group (ie, raw view) indicated that those in the mHealth and paper diary groups reported symptom improvement at months 3 and 6 from baseline. It is important to note that individuals with T2D in the study had greater symptom burden and poorer physical functioning at baseline than the general US population. The PROMIS *P* values were >.05; however, positive trends were noted in both intervention groups (mHealth and paper diary) in the middle (month 3) and end (month 6) of the study. Our results found that the mean scores of our participants for most domains were poorer than those of the US reference population. This suggests that overweight or obese individuals diagnosed with T2D have higher symptom burden and poorer functioning compared with healthy individuals. The results from our study support the use of the PROMIS questionnaires to provide clinicians and researchers with a benchmark for assessing the overall need for disease management and determining the success or challenges of an intervention.

Similar results have been reported in a cross-sectional study among patients diagnosed with T2D, where patients reported higher levels of pain and fatigue that were closely related to sleep disturbance [15]. Another cross-sectional study reported that patients who were very active in their self-care related to T2D had lower depression, better social outcomes, and better physical function [16]. To address the missing items from our study, we looked at the study conducted by Paz et al [17] that estimated the readability of the PROMIS questionnaires to assess their comprehensibility in a sample of African American and Latino older adults (aged ≥65 years). The authors reported that the participants had challenges in readability, comprehension, and interpretation of PROMIS items. The authors further reported that the study participants had limited educational attainment and socioeconomic status (similar to our study) and may experience cognitive decline from aging, chronic diseases, and possible polypharmacy, which could have contributed to our findings of missing items.

### Strengths and Limitations

The PROMIS instruments chosen for the study offer an opportunity to explore a variety of health concerns in patients with T2D, which may not have time for discussion during a standard clinic visit. The participation retention rate of 92% at 6 months supports the idea that participants are interested in and accepting of self-monitoring behavioral lifestyle interventions.

Our pilot study has several limitations. It is important to note that the study population included individuals who were overweight or obese and had T2D, predominantly included African American people, and mostly included individuals lacking health insurance and having a lower socioeconomic status. At baseline, they had lower PROMIS scores than the mean score of the general US population (mean 50, SD 10), and their scores stayed low throughout the study. The participants were those seeking care at a diabetes education center and thus may not be representative of the general population. In addition, the pilot study was not designed to detect statistical significance, as the study was a feasibility study.

### Conclusions

Our pilot study provides evidence for the feasibility of using the PROMIS questionnaires to measure patient-reported outcomes among overweight or obese individuals diagnosed with T2D. It is important to note that individuals with T2D in this study started out with greater symptom burden and poorer physical functioning at baseline compared with the general US population. The results from our study support the use of PROMIS questionnaires to provide clinicians and researchers with a benchmark for assessing the overall need for treatment and determining the success or challenges of the intervention. The *P* values of the PROMIS-57 and PROMIS-GH were >.05 (not significant); however, positive trends were noted in both intervention groups (mHealth and paper diary) in the middle (month 3) and end (month 6) of the study.

Future studies should consider using PROMIS computerized assessment testing that may be associated with higher completion rates and may reduce respondent burden by limiting the number of questions (fixed length) that participants need to answer. Future directions include (1) development and validation of a T2D-specific PROM that combines persons with similar clinical characteristics and risks for complications to identify treatment needs and (2) integration of these patient-reported outcome tools into routine patient care and research studies.

## Abbreviations

GH: global health
GMH: global mental health
GPH: global physical health
mHealth: mobile health
PROM: patient-reported outcome measure
PROMIS: Patient-Reported Outcome Measurement Information System
T2D: type 2 diabetes
